# Qualitative Study to Understand Pediatric Hospitalists and Emergency Medicine Physicians’ Perspectives of Clinical Pathways

**DOI:** 10.1097/pq9.0000000000000270

**Published:** 2020-03-25

**Authors:** Kimberly O’Hara, Melisa Tanverdi, Jennifer Reich, D. David Scudamore, Amy Tyler, Leigh Anne Bakel

**Affiliations:** From the * Department of Pediatrics, University of Colorado School of Medicine, Aurora, Colo.; † Department of Sociology, University of Colorado, Denver, Colo.

## Abstract

**Introduction:**

Healthcare costs are rising, and clinical pathways (CPW) are one means to promote high-value care by standardizing care and improving outcomes without compromising cost or quality. However, providers do not always follow CPW, and our understanding of their perceptions is limited. Our objective was to examine pediatric hospital medicine (PHM) and pediatric emergency medicine (PEM) physician perspectives of CPW.

**Methods:**

We conducted semistructured, in-depth, one-on-one qualitative interviews with PHM and PEM physicians between February 2017 and August 2017. Interviews were audio-recorded, professionally transcribed, and accuracy verified. Using an inductive analytic strategy, we systematically coded the data to identify themes.

**Results:**

We interviewed 15 PHM and 15 PEM physicians. These providers identified many benefits and limitations of CPW, which positively or negatively impact resource utilization, communication, education of personnel, patients, and families, as well as practice behaviors and attitudes. Perceived benefits included (1) reduction of unnecessary utilization, (2) standardization of care, (3) improved communication, (4) education of oneself and others, and (5) confidence and validation when actions align with CPW. Limitations of CPW were (1) resource utilization for revisions, updates, and dissemination; (2) “tunnel vision” and cognitive biases; (3) loss of autonomy; (4) prescriptive medicine; (5) information overload; (6) pressure to adhere; and (7) guilt if actions do not align with CPW.

**Conclusions:**

CPW are tools with advantages and disadvantages that are used and viewed differently by providers. Such insight into how physicians perceive CPW may help to optimize hospital improvement work and enhance high-value care.

## INTRODUCTION

Clinical pathways (CPW) aim to integrate evidence into clinical practice and optimize patient outcomes while improving efficiency.^1^ Although healthcare costs in the United States are increasing at an unsustainable rate, physicians are responsible for directing over 85% of healthcare spending.^2–4^ CPW are one means to tackle this economic problem as they guide evidence-based medicine in more than 80% of hospitals in the United States as of 2003.^5^ The need to practice high-value care, particularly by intervening at the provider level and understanding their perspectives, experiences, and practice behaviors regarding CPW, is critical.

CPW are tools that standardize care processes and improve outcomes without increasing the cost or compromising quality.^1^ In 2012, a Cochrane systematic review of CPW revealed that CPW decrease hospital complications and improve documentation without increasing length of stay or cost.^1^ In 1 study, the use of a pathway for patients with diabetic ketoacidosis resulted in improved documentation of severity indices. The residents found this pathway to be user-friendly, informative, and a tool that positively impacted management.^6^ However, literature exploring other providers’ views of CPW for different disease processes or CPW, in general, is limited. To our knowledge, no studies have evaluated specifically pediatric hospital medicine (PHM) or pediatric emergency medicine (PEM) physicians’ perceptions of CPW.

By 2017, our institution had created 60 CPW, which are used to translate national guidelines and the latest evidence into the local context. CPW are typically developed by clinical improvement teams with multidisciplinary members, such as primary care providers, subspecialists, pharmacists, nurses, and caregivers.^7^ Although our institution continues to develop and disseminate these tools, how frontline providers perceive and apply CPW in clinical practice remains unclear.

To optimize hospital systems’ improvement work and provide high-value care, hospital leadership must understand more clearly how frontline providers view and apply CPW in their daily practice. We aimed to conduct a qualitative study to examine PHM and PEM physician perspectives of CPW. By gaining insight into how these providers perceive CPW, hospital leaders can enhance CPW to conduct meaningful quality improvement (QI) work and further improve patient outcomes efficiently and safely.

## METHODS

### Qualitative Approach

We collected qualitative data during one-on-one interviews with attending physicians at a large pediatric academic health center. Our qualitative descriptive approach consisted of thematic analysis, a “method for identifying, analyzing, and reporting patterns (themes) within data.”^8^ We applied thematic analysis methodology to the transcribed interviews to gain an in-depth understanding of CPW perspectives by identifying codes, themes, and further interpretation as outlined below.^9^

### Setting

We conducted interviews at a free-standing, quaternary Children’s Hospital affiliated with a University, which has approximately 161,000 emergency and urgent care visits and 15,000 inpatient admissions annually.^10^ Participants were physicians who worked at this Children’s Hospital and/or at 1 of the 8 affiliated, local community hospitals in rural, suburban, and urban areas. These physicians had access to the same CPW regardless of the working site via the electronic medical record as well as internal and external websites.

Our institution’s CPW program was implemented in 1994, and we renovated the program starting in 2015 as described by Pugh-Bernard et al.^11^ Until 2018, our healthcare providers referred to CPW as “guidelines,” at which time the institution officially changed the term “clinical care guidelines” to CPW. Our CPW have always aligned with the 4 criteria for CPW as defined by Lawal et al^12^: the intervention [CPW] “(1)…was a structured multidisciplinary plan of care; (2)…was used to translate guidelines or evidence into local structures; (3)…detailed the steps in a course of treatment or care in a plan, pathway, algorithm, guideline, protocol, or other “inventory of actions” (ie, the intervention had time frames or criteria-based progression); and (4)…aimed to standardize care for a specific population.” CPW are updated at least every 4 years to ensure continuous improvement and to reflect the latest evidence.^7^ Implementation studies of various CPW at our institution have demonstrated improvements in evidence-based care in the inpatient and emergency department settings.^13–19^

### Recruitment

All physician faculty members of the PHM and PEM sections were invited to participate by email. If a physician did not respond to the first invitation, we sent 2 follow-up emails. Participation was voluntary, and subjects did not receive any financial or other incentives to participate. Our institution’s review board approved the study, and participants provided written consent before their interview.

### Data Collection

We developed a semistructured interview guide designed to elicit perceptions of CPW. Sample questions include, “What are your views of CPW in general?” and “In your experience, how have CPW changed over time?” Two investigators (a hospitalist and a PEM fellow) conducted semistructured, in-depth, face-to-face interviews with PHM and PEM physicians between February 2017 and August 2017. These investigators previously knew the participants professionally as colleagues. As practicing physicians, they had the experience needed to understand the content and underwent training by a qualitative research expert to learn how to conduct interviews properly.

Interviews were audio-recorded, professionally transcribed verbatim, and accuracy verified by reading the transcripts while listening to the recordings. We uploaded the transcripts to Dedoose Version 8.2.14 (SocioCultural Research Consultants), a platform for qualitative research that was used to track codes.^20^ We also collected the characteristics of providers, including gender, academic titles, and years in practice.

### Data Analysis

Before the first interview, we decided conducting 30 interviews would be feasible and aimed to divide them between the PHM and PEM sections. Saturation, the point at which no new information is learned, was achieved shortly before the 30th interview. However, we completed and coded all 30 interviews to ensure all perceptions were uncovered. Data collection and analysis occurred simultaneously. Preliminary analysis began after the first interview and continued during subsequent interviews informing ongoing data collection; the 2 investigators who conducted the interviews developed thematic codes using an inductive analytic strategy.^9,21^ After data collection was complete, the data were systematically coded by 2 additional investigators to ensure quality. Four investigators, the 2 who performed interviews and initial coding plus the 2 additional investigators met to finalize codes and identify subthemes and themes.

## RESULTS

### Demographics

We interviewed 15 PHM and 15 PEM physicians; 57% were women, and 43% were men. Regarding academic appointments, 16% were instructors, 7% were senior instructors, 50% were assistant professors, and 27% were associate professors. The mean years in practice since residency was 9 (SD = 6.1).

### Findings

**Fig. 1.**
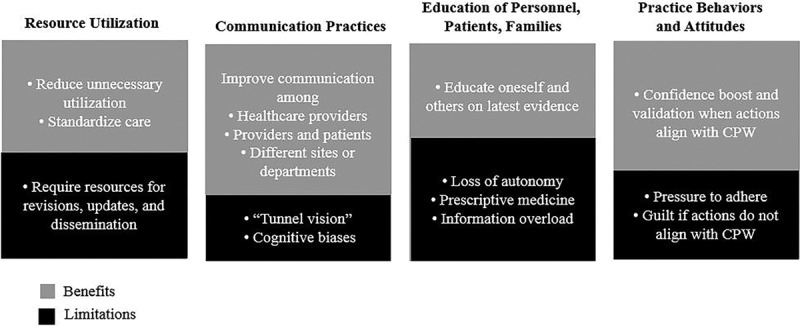
Conceptual model of the benefits and limitations of Clinical Pathways that emerged from interviews with physicians.

Both PHM and PEM physicians identified benefits (Table 1) and limitations of CPW (Table 2). Data analysis revealed a central theme that physicians consider CPW to be advantageous and disadvantageous, impacting resource utilization, communication practices, education of personnel, patients, and families, as well as practice behaviors and attitudes. Within each of these categories or subthemes, CPW have both positive and negative aspects or qualities, which varied by the provider (Fig. 1). These perceived benefits and limitations might consequently influence how one adheres to or applies CPW in the care of patients.

**Table 1.**
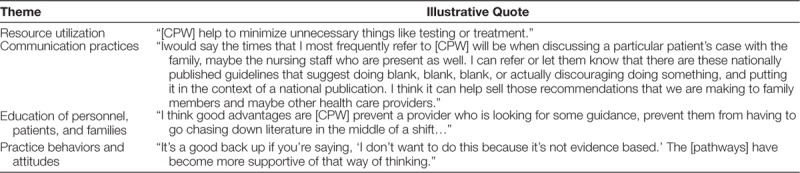
Additional Illustrative Quotes from Physicians Regarding Clinical Pathway Benefits

**Table 2.**
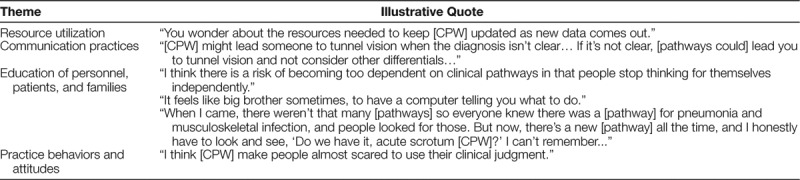
Additional Illustrative Quotes from Physicians Regarding Clinical Pathway Limitations

### Benefits of CPW

#### Resource Utilization

The first benefit expressed by participants was that CPW encourage high-value care by decreasing unnecessary utilization of healthcare resources, emphasizing the evidence, and standardizing care. One provider commented, “[CPW] standardize care around areas where evidence is clear and eliminate unnecessary variation.” Although healthcare costs are escalating, physicians recognized that CPW increase quality and reduce costs through promoting resource stewardship, practicing evidence-based medicine, and eliminating variation.

#### Communication Practices

Participants emphasized that CPW improve communication among team members and different sites to ensure that everyone is on the same page. One provider reported, “I think you have a lot of different people working on the team… [CPW] help streamline communication and what the intervention is.” Also, physicians remarked that they use CPW to explain their management to patients and families. One individual stated, “…[CPW] give us a lot of leverage to explain our decision-making with parents. …If [parents] ask a question about wanting viral testing or chest x-rays, I think we’re very well armed with our [CPW] and data to explain to them why we don’t think it’s indicated.” CPW not only improve communication among team members but facilitate conversations with patients and families regarding plans of care.

#### Education of Personnel, Patients, and Families

Along with ensuring a shared understanding of the plan, CPW have an instrumental role in educating patients, families, trainees, and clinicians. One physician explained, “[CPW] prevent a provider who is looking for some guidance, prevent them having to go chasing down the literature in the middle of a shift…” Participants expressed that they use CPW to educate trainees on the literature and their clinical decision-making. A participant explained, “It’s easy to educate people on the pathophysiology and the clinical care using the [CPW] as a template. So you can sit with a medical student and say, ‘We chose this particular antibiotic in the pathway because…’”

In addition to using CPW as an educational tool, physicians noted that CPW are advantageous for providers with less clinical experience. One noted, “[CPW] are good for guidance for younger trainees or for people who have been out for a shorter period of time who don’t necessarily have that breadth of experience.” CPW are considered a valuable tool for providers new to an institution as well. Participants stated that new faculty or trainees use CPW to learn about their local system; for example, CPW provide information on how to approach certain diagnoses, including which subspecialist to consult.

#### Practice Behaviors and Attitudes

CPW offer the additional benefit of providing practice validation, fostering confidence, and affirming clinical decision-making skills. As one stated, “This is one of the benefits of [CPW]. It gives people… more ability to say, even though [that provider gave] five albuterols, I’m going to be fine doing nothing.” For physicians who work in more isolated settings or at hospitals without subspecialists available for in-person consults, CPW validate that one is practicing according to the latest literature and similar to colleagues. A physician explained, “I think it’s nice to validate sometimes what you already do in practice, knowing that a group of people spent a lot of time reviewing the evidence and came to the same conclusion of how you’re practicing.” Participants recognized they feel supported and more confident when they adhere to CPW, and their practice aligns with the recommendations.

### Limitations of CPW

#### Resource Utilization

Physicians noted that CPW development and implementation require institutional investment. A multidisciplinary working group is often assembled to review the literature and create a pathway applicable to the institution. To be trusted and used, CPW must also be revised and updated with the latest evidence promptly. One provider described, “I think the disadvantages are when they’re not revised and reviewed. Because [CPW] can become outdated… and then… people don’t follow them, and then they don’t believe in them anymore.” Physicians also reported that the CPW that are better advertised and supported by leadership are recognized and utilized more. Consequently, providers may not be aware of certain CPW if the pathways lack the institutional support or resources to disseminate them adequately.

#### Communication Practices

CPW aim to improve communication, but some providers felt they also limit conversations by creating “tunnel vision.” Participants emphasized that CPW can discourage providers from considering or discussing alternative diagnoses with others. One physician stated, “I think [CPW] can put you at risk of some of the biases, cognitive biases… anchoring particularly. I think people do not think outside the box once a patient has a certain diagnosis that puts them down a care path.” Once a patient is associated with a certain pathway, physicians noted that conversations about other diagnoses become limited or do not occur.

#### Education of Personnel, Patients, and Families

Participants expressed concern that CPW encourage providers to adhere to an algorithm or an outlined plan, which can stifle one’s education by limiting critical-thinking skills and autonomy. CPW lead to “prescriptive medicine” where care may be simplified too much; this perspective was emphasized specifically by those with more experience, usually associate professors. As one explained,"I think there's something nice to be said for being able to explain to a parent, 'Here's why we're doing something, our hospital did a huge review on this, and we all agree." Also, physicians noted that inclusion and exclusion criteria for CPW are often not recognized due to a lack of knowledge. Consequently, patients may be started on a pathway when it was not intended for that specific population, and thus, the right care is not provided.

Participants shared another disadvantage of CPW is “information overload,” where the number and length of pathways are perceived to be increasing over time. Providers find it challenging to remain up-to-date on which pathways exist and are unable to educate oneself on the content. One participant explained, “I mean every month it seems like there’s a new [CPW]. And it’s sometimes hard to keep track of the information...” Participants emphasized that CPW are more useful educational tools when they can be accessed quickly and navigated efficiently during a busy shift.

#### Practice Behaviors and Attitudes

Participants expressed both their colleagues and their nonadherence to CPW can result in a range of emotions from fear to frustration. Physicians reported pressure to abide by CPW; as 1 physician described, “There’s pressure to follow [CPW]. And it also makes people look back at your care and wonder, ‘Why did you do that, why are you not following [the CPW]?’” This pressure to comply results in providers describing feelings of guilt when nonadherent, which can prevent high-quality care and create conflict within a team. A participant explained, “I had a case where a child was initially admitted under bronchiolitis, and when I saw the child, they were not responding like a bronchiolitic. I actually needed to treat this child like an asthmatic, and the Respiratory Therapist flat out refused… It was hard to get her to switch from [the] bronchiolitis [pathway] to an asthma pathway...” Such effects on practice behaviors and attitudes can lead to low-value care. Lastly, although nearly all physicians described both positive and negative experiences with CPW, some expressed ambivalent perceptions, explaining that they rarely rely on CPW and thus practice independent of CPW.

## DISCUSSION

The knowledge gained in this study helps to advance our understanding of how CPW are valued, applied, and impacting clinical care in the emergency department, urgent care, and inpatient settings. CPW are tools perceived and used differently by different physicians. Depending on the provider, CPW are perceived to positively or negatively impact resource utilization, communication practices, education of personnel, patients, and families, as well as practice behaviors and attitudes. For example, one sees CPW as a tool for safely doing less, whereas another believes CPW lead to cognitive biases, and consequently, providers miss things. One sees CPW as validation and support for actions, whereas another considers CPW as forcing their hand. Knowledge and recognition of these perceived benefits and limitations are necessary to maximize CPW adherence when appropriate and to improve patient care.

Prior studies have reinforced CPW effects on education, including pathways that help residents apply evidence-based medicine and work with other team members.^22,23^ The results from our study expanded on this finding from a physician’s viewpoint that CPW serve as educational tools not only for trainees but for all team members, new providers, and patients/families. We also learned the potential benefits of CPW and further detailed their limitations regarding resources, communication, practice behaviors, and attitudes. Those responsible for CPW or who develop CPW as QI interventions should consider such physicians’ perceptions. For example, when creating CPW, they should aim for shorter lengths and algorithms that are easy to navigate. The value of marketing or a campaign to advertise new CPW cannot be underestimated. Additionally, education regarding the potential cognitive biases associated with CPW use is critical.

CPW are one of many different implementation strategies, each with various ratings of importance and feasibility.^24^ For those charged with creating and implementing CPW, understanding how best to combine CPW with other implementation strategies is necessary as this may impact perceptions, acceptance, and applicability of CPW. Implementation strategies, such as identifying early adopters, local consensus discussions, capturing and sharing local knowledge, and developing educational materials, are key considerations to abate the limitations of CPW perceived by our PHM and PEM physicians.^24^ This study identified barriers and facilitators related to CPW, which must be addressed to optimize QI work, clinical efficiency, and patient outcomes.

Limitations of the study include it was a single-center study affecting its generalization. However, our providers work at multiple different hospitals, from a quaternary Children’s Hospital to community hospitals of varying sizes and locations. Another limitation is this study elicited perceptions of physicians and cannot speak to how these views affect true practice or CPW adherence as we did not measure behaviors at the bedside. Because interviews were conducted by colleagues, it is possible that these perceptions were biased if participants wanted their responses to appear more acceptable to their peers. Assurances to limit such bias were made by training the interviewers, interviewing multiple physicians, and having 4 different investigators analyze the data. Last, we deliberately interviewed physicians only as these providers have more similar training, privileges, and responsibilities. However, we can learn more from engaging other team members involved in delivering patient care.

Future studies are needed to address the above limitations and better understand if one’s perceptions align with clinical practice. Mixed method studies to measure practice behaviors quantitatively and determine if certain perceptions are associated with those who ordered more or fewer interventions are necessary. Elucidating additional perspectives from trainees, other subspecialists, and patients/families may reveal how best to engage all stakeholders in CPW development and implementation. Such heightened understanding would help organizations effect positive behavioral change to improve patient outcomes further.

At a time when healthcare expenditures are escalating, and physicians are at the front line of ordering interventions, the information gathered from our PHM and PEM providers elucidates perceptions and utility of CPW, which hold the promise of providing evidence-based, high-value care. Physicians view CPW differently, and thus, how they implement these tools in clinical practice varies. Such insight into how physicians perceive CPW may help to optimize hospital systems improvement work, reduce healthcare waste, and provide high-quality care.

## DISCLOSURE

The Section of Pediatric Hospital Medicine at the University of Colorado School of Medicine provided financial support for a Professional Research Assistant to transcribe interviews. The authors have no financial interest to declare in relation to the content of this article.
